# Supplementary material for: MetaRibo-Seq measures translation in microbiomes
Source: Nat Commun. 2020 Jun 29;11:3268. doi: 10.1038/s41467-020-17081-z (PMC7324362; doi:10.1038/s41467-020-17081-z)
Supplement: Supplementary file 10 — Supplementary Data 7 [file 41467_2020_17081_MOESM10_ESM.zip › File2/Confidence_High_Taxonomy/84859_out.krona.html]

Javascript must be enabled to view this page.

members
magnitude
magnitudeUnassigned
count
unassigned
taxon
rank

84859\_out

3

2
superkingdom
3

1239
3
phylum

186801
class
3

order

SRS098571\_contig\_number\_contig-100\_2100.370630
1
3
186802

541000
2
family

946234
genus
2

2
species

SRS013687\_contig\_number\_contig-100\_5295.158738SRS023914\_contig\_number\_contig-100\_1982.158443
1193534
